# Infection by Hepatitis Delta Virus

**DOI:** 10.3390/v12060648

**Published:** 2020-06-16

**Authors:** John M. Taylor

**Affiliations:** Fox Chase Cancer Center, Philadelphia, PA 19111, USA; john.taylor@fccc.edu

**Keywords:** hepatitis delta virus, hepatitis B virus, coinfection, superinfection, monoinfection, envelope proteins, pseudotypes

## Abstract

Hepatitis delta virus (HDV) and hepatitis B virus (HBV) are blood-borne viruses that infect human hepatocytes and cause significant liver disease. Infections with HBV are more damaging when there is a coinfection with HDV. The genomes and modes of replication of these two viruses are fundamentally different, except for the fact that, in nature, HDV replication is dependent upon the envelope proteins of HBV to achieve assembly and release of infectious virus particles, ones that use the same host cell receptor. This review focuses on what has been found of the various ways, natural and experimental, by which HDV particles can be assembled and released. This knowledge has implications for the prevention and treatment of HDV infections, and maybe for an understanding of the origin of HDV.

## 1. Introduction

Hepatitis delta virus (HDV) was discovered in 1977, with the observation of a novel antigen in the liver of patients already infected with the hepatitis B virus (HBV) and exhibiting symptoms of a more damaging infection [1]. Initially, this antigen, the delta antigen, was considered to be the protein of a more aggressive form of HBV. However, it was soon found that it is produced by a separate virus, HDV, one that uses HBV as its helper virus [2]. Since that time, much has been learnt about the replication of HDV and HBV, its natural helper virus. Additionally, after testing stored sera samples for delta antigen, it was found that ever since 1934, a fraction of HBV-positive patients in the Amazon basin were also positive for the delta antigen [3].

## 2. Coinfection, Superinfection, and Monoinfection

In patients, both HBV and HDV are exclusively infections of hepatocytes in the liver. As currently understood, both viruses use the HBV envelope proteins to attach to the same hepatocyte receptor, sodium taurocholate cotransporting polypeptide, NTCP [4]. Both viruses replicate in these hepatocytes, and new infectious particles are released into the bloodstream. All HDV infections begin with contaminated blood or a blood product. Two kinds of human infection are distinguished: “coinfection” and “superinfection” [5].

An HDV coinfection is defined as occurring in an individual not previously exposed to either HBV or HDV. However, there are many individuals that have already been infected with HBV in the absence of HDV. Such infections can progress to becoming chronic, defined as lasting at least six months, but possibly for decades. It is variously estimated that globally, more than 240 million are chronically infected [6]. When such an individual is exposed to HDV, it is referred to as a superinfection.

The various outcomes of co- and superinfections, as reviewed in detail elsewhere [5], can overlap, but on average, they are different. Both can cause an acute infection (less than 6 months) that can lead to fulminating hepatitis; however, this outcome is more frequent for superinfections. Both can cause a chronic HDV infection, but this is more likely for a superinfection. A coinfection is less likely to do this because chronic HDV also requires establishing a chronic HBV.

In patients, chronic HBV infections are associated with continued liver damage and an increased risk of progressing to cirrhosis and liver cancer. When HDV is also present, there is a more rapid progression to cirrhosis [7].

Societally, patients with indications of liver damage are screened for HBV markers but not always for HDV. However, with increased testing and/or more sensitive assays for HDV, it would seem that, on average, 14% of chronic HBV patients are also chronically infected with HDV. This leads to estimates of 70 million infections worldwide [5,8]. A dramatic exception is that amongst HBV-positive intravenous drug users, the incidence reaches 70%.

An additional unique complication to the infection process is that infectious HDV can enter and replicate its genome in the absence of HBV. Such a monoinfection can survive for at least 6 weeks, and yet, with a subsequent HBV infection, infectious HDV can be assembled and released. This process has been demonstrated experimentally in mice harboring human hepatocytes [9]. It is supported by human studies, where some hepatocytes can demonstrate HDV antigen in the absence of detected HBV markers. While HDV entry and replication in the absence of HBV is possible, although nonproductive, it may yet make changes in the hepatocytes, namely, the induction of interferon response genes that could contribute to disease in the host [10].

## 3. HDV Genome Replication

In 1986, three reports, using different strategies, showed that the genome of HDV is not just a small RNA of about 1700 nucleotides, but one with a circular conformation [11,12,13]. Unlike HBV, this RNA replicates without a DNA intermediate, by RNA-directed RNA synthesis, producing an exactly complementary RNA circle, designated as the antigenome. Both genome and antigenome, via about 70% intramolecular base-pairing, will fold into a rod-like structure. Moreover, each RNA contains a single small domain that will act as a ribozyme to bring about site-specific RNA cleavage [14,15]. 

Later studies provided many more details of HDV genome replication, including the presence of a less abundant, smaller linear RNA of antigenomic polarity, 5’-capped and 3’-polyadenylated, that contains the open reading frame for translation of the delta antigen. During replication, there were two species of delta antigen, and it was shown that the small 24-kDa species is essential for genome replication [16], while the large 27-kDa species arises via site-specific post-trancriptional RNA editing [17] and contains a C-terminal region not present on the small antigen. The large form is an inhibitor of the replication supported by the small form [16], but it is also an essential part of the virus assembly process [18]. Additionally, this requirement depends upon a post-translational site-specific farnesylation of the C-terminus [19]. Both small and large forms are RNA-binding proteins and able to provide stabilization of the RNA circles [20]. The essential role of the small form might include facilitating redirection of host DNA-directed RNA polymerases to transcribe HDV RNA templates [21,22], facilitation of ribozyme activity [21], and regulation of delta antigen mRNA levels [23]. The specific roles of the two antigens are linked to their intracellular localization and the presence or absence of post-translational modifications [21].

As mentioned earlier, HDV infection and genome replication can be initiated in human hepatocytes in the absence of HBV [9]. Moreover, experimentally, HDV genome replication can be initiated via various transfection procedures, all in the absence of HBV [24,25]. Furthermore, under experimental conditions, HDV replication can be initiated in cell types other than hepatocytes and in nonhuman cells. However, assembly and release of infectious HDV normally need HBV help.

## 4. HDV Assembly

HBV assembly is both a complex and inefficient process [26]. An intermediate step is the formation of a nucleocapsid (or core), a regular icosahedral structure, composed of multiple copies of a capsid protein and containing an HBV RNA, called the pregenome, already in the process of being copied into DNA by a viral reverse transcriptase. This nucleocapsid structure, in later stages of assembly, makes interactions with HBV envelope proteins embedded in host intracytoplasmic membranes. However, the assembly process is grossly inefficient in that at least a 100-fold excess of empty noninfectious particles are released [26].

As mentioned earlier, HDV genome replication is totally different from that of HBV. Moreover, of more relevance here, HDV does not produce a regular nucleocapsid structure with its genome inside. HDV again appears to be fundamentally different. That is, the delta antigens form multimers, and the HDV RNA wraps around such multimers. In fact, some studies suggest the multimers may be predominantly octamers of the delta antigen and that the genomic RNA, which is partially double-stranded, wraps around the octamers in a way analogous to how double-stranded host DNA wraps around histone octamers [27]. Moreover, a recent study supports the interpretation that such histone-mimicry drives the recruitment of chromatin remodelers leading to the recruitment of RNA polymerase II [22].

HBV replication produces three related envelope proteins, each sharing their C-terminal regions. They are designated according to size, as large, middle, and small (L, M, and S) [28]. L contains a unique N-terminal domain essential for attachment to the hepatocyte receptor, NTCP [4]. HBV assembly typically uses all three proteins, but the process is inefficient in that infection produces relatively huge amounts on empty noninfectious particles, with relatively large amounts of just the S protein [26]. HDV assembly appears to exploit this inefficiency, yet as with HBV, only particles containing the L protein can infect hepatocytes via interaction with NTCP [4].

In experimental situations, expression of the HBV envelope proteins, in the absence of HBV replication, is sufficient for the assembly of infectious HDV [29]. The M protein is not essential for assembly or infectivity [30]. In addition, the S protein is sufficient for assembly, but the particles are noninfectious [30].

In chronically infected patients HBV patients, there can be found integrated regions of HBV DNA [31]. Furthermore, some integrations can lead to the expression of the HBV envelope proteins. Thus, it is not surprising that cell lines derived from HBV-associated liver tumors and expressing HBV envelope proteins, when transfected to support HDV genome replication, will release infectious HDV particles [32].

## 5. Alternative Helpers for HDV Assembly

With time, it has been shown that many animal species are infected by HBV-like viruses. All are now classified as the family *Hepadnaviridae* [33]. There are five genera, with HBV in the founding mammalian genus, *Orthohepadnaviridae*. Those specific to birds are separately classified as *Avihepadnaviridae*. Several of these family members have been tested for their ability to support HDV assembly. An early and striking success was with the woodchuck hepatitis virus, WHV [34]. Up to 20% of woodchucks in one geographic location in the US are chronically infected with this virus, and after 1–2 years, this typically leads to hepatocellular carcinoma [35]. If a chronically infected animal is further infected experimentally with a human HDV, the HDV produces an extreme acute infection, often leading to fulminating hepatitis [34]. We would call such HDV as pseudotyped with WHV envelope proteins. Moreover, such a pseudotyped virus can be experimentally transmitted back into primates.

Bats are another example of an animal where HBV-like species can sometimes be found. In one experimental study, the envelope proteins of a bat hepadnavirus were found to be able to support the assembly and release of pseudotyped particles from cells replicating HDV. The particles were infectious on primate hepatocytes, and the evidence supported the interpretation that the entry was via NTCP, the known receptor protein for human HBV and HDV [36].

Among various avian species, infections by what are classified as the genus *Avihepadnaviridae* can be found. However, no success has been detected in achieving HDV assembly by such viruses.

Recently, a careful experimental study tested non-hepadnavirus envelope proteins for their ability to produce HDV pseudotypes [37]. Several of these viral proteins produced efficient egress of RNA-containing particles, and some particles were found to have infectivity with the host specificity altered to that of the provided envelope proteins. Striking was that one of the non-hepadnaviruses successfully tested was the hepatitis C virus, and it was further shown that this virus facilitated the spread of HDV in mice bearing human hepatocytes. This supports the speculation that in patients, HCV alone could support HDV assembly and spread, or at least provide assistance, in addition to that from HBV.

## 6. HDV-Like RNAs

Since 2018, three studies have reported HDV-like RNAs in a variety of nonhuman animals, vertebrates and invertebrates [38,39,40]. Common features of this similarity include that these RNAs are of about the same size, of a circular conformation, and have the ability via extensive intramolecular base-pairing to form a rod-like structure. These RNAs, although dramatically different in nucleotide sequence to human HDV, are predicted to have an open-reading frame analogous to a delta antigen. Additionally, they are predicted to have two HDV-like ribozymes, although functionality has not yet been tested. Some of these studies suggest there is RNA replication. Moreover, experimentally, when envelope proteins from certain other viruses are provided, there can be spread of the RNA to new host cells [38].

These important studies have opened a new door in terms of understanding the origin of HDV RNA. That is, they lead to novel speculations that a replication-competent HDV-like RNA arose from a nonhuman source and may have achieved assembly and infectivity in hepatocytes of patients already infected with HBV.

## 7. Prevention and Treatment

Because of the shared envelope proteins and shared host receptors, both HBV and HDV infections can be prevented by vaccination. In contrast, the treatment of chronic HDV infections is difficult [5]. One strategy uses peptides that mimic the site on the large HBV envelope protein needed for interaction with the host receptor [41]. This blocks the entry of both HBV and HDV. A similar strategy might be to inhibit the NTCP itself [42]. A different strategy uses specific nucleic acid polymers (NAPs). These inhibit both virus entry and secretion but can have serious side-effects [43]. Another strategy, applicable to HDV only, is to use lonafarnib, an inhibitor of the farnesylation of the large delta antigen. This works experimentally since this modification is essential for the release of HDV [44]. Preliminary clinical results using this strategy have been reported [45,46]. Other HDV-specific inhibitors have been tested experimentally [5]. While the use of interferons to treat chronic HDV is marginally successful clinically (in up to 30% of patients), it is still considered as part of combination therapies [5].
